# The Role of Counteranions in Solution Deposition of ZnS Thin Films on GaAs

**DOI:** 10.1021/acs.inorgchem.4c03424

**Published:** 2024-12-11

**Authors:** Noy Zakay, Shlomo Rand, Alexander Rashkovskiy, Nitzan Maman, Vladimir Ezersky, Yuval Golan

**Affiliations:** † Department of Materials Engineering, Ben-Gurion University of the Negev, Beer-Sheva 8410501, Israel; ‡ Ilse Katz Institute for Nanoscale Science and Technology, Ben-Gurion University of the Negev, Beer-Sheva 8410501, Israel

## Abstract

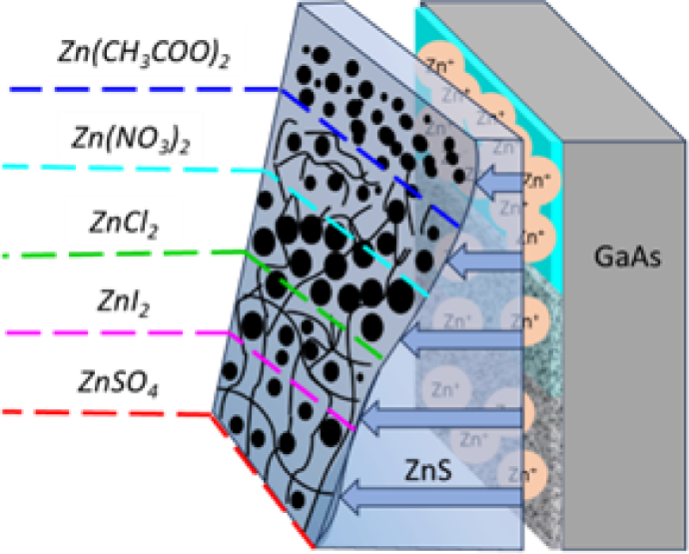

Using 5 commonly employed precursors for Zn^2+^ cations, ZnSO_4_, ZnI_2_, ZnCl_2_, Zn(NO_3_)_2_, and Zn(CH_3_COO)_2_, we demonstrate the effect of counterions on ZnS thin film formation. We show that both film nucleation and growth stages are strongly dependent on the Zn^2+^ precursor type. We systematically studied the mechanisms of thin film deposition on GaAs(100), including solution–substrate interactions, Zn^2+^ ion adsorption on the substrate, and nuclei formation as well as stress accumulation during film growth. It was shown that the early stages of film formation play a key role in the subsequent growth kinetics and resulting film quality. X-ray photoelectron spectroscopy, supported by contact angle measurements, transmission electron microscopy, and energy-dispersive spectroscopy showed that increasing the degree of surface oxidation and passivation at the early stages of ZnS film formation inhibits solution desorption. Finally, the previously reported mechanism of crack formation is explored through an interrupted growth series of ZnS films deposited from solutions with different Zn^2+^ precursors.

## Abstract 2

The role of the source precursors for zinc cations in solution deposition of ZnS thin films on GaAs was investigated. ZnSO_4_ or ZnI_2_ are precursors for rapid deposition of a continuous ZnS wetting layer. In cases where the substrate surface needs to be maintained in its native state, the Zn acetate precursor is preferred, albeit the slower growth rate. Dominant nucleation in solution is in close proximity to the substrate when using the ZnCl_2_ precursor.

## Introduction

Zinc sulfide (ZnS) is an important VI-II semiconductor compound with a wide direct band gap of 3.65 eV.^1−3^ This material holds promise for implementation in optoelectronic devices, including blue light-emitting diodes,^4^ electroluminescent devices, and photovoltaic cells.^5−8^ It is also utilized in the detection, emission, and modulation of visible and near-ultraviolet light.^9−11^ Density functional theory calculations were employed to predict the band structures of the two common phases of ZnS, the cubic zincblende phase, which is only slightly more favorable compared to the hexagonal wurtzite phase. The results showed that in both phases, the minimum energy of the conduction band is more dispersive than the maximum of the valence band, implying superior electron mobility compared to hole mobility. Additionally, the *p-*electrons in the valence band are strongly bonded to sulfur atoms in the lattice, further impeding hole movement.^12^ ZnS shares similar properties with CdS, having the same zincblende or wurtzite crystalline structure. These semiconducting compounds can emit light when excited, making them useful in optoelectronic devices in applications, such as photocatalysis. The band gap of CdS is narrower by 1.0–1.2 eV compared to that of ZnS, with reported values around 3.4–3.6 eV and a strong blue shift due to the quantum size effect. ZnS is generally considered to be an environmentally friendly alternative, making it a useful potential replacement for toxic CdS as a buffer layer in solar cells.^13^

Chemical solution deposition of semiconductor thin films (CD), also known as chemical bath deposition (CBD), is a widely studied and cost-effective technique that enables deposition at low temperatures without advanced environmental requirements like vacuum or plasma.^14−16^ It is commonly used for depositing high-quality aqueous-based semiconductor thin films.^17−20^ ZnS has been previously deposited as thin films using CD,^21−26^ with several reports notably showing cracks in the films.^24,27^

The contact angle observed on the substrate, and on the evolving thin film during deposition, is an important parameter that provides insights into the wetting behavior and surface characteristics of the deposited material^28^ and can be affected by various factors such as substrate surface treatment, surface oxidation, and passivation.^29^ In the case of ZnS thin films, the contact angle is also influenced by factors like the deposition method, film thickness, and surface modification.^30^

It is well-established in the scientific community that the choice of precursor materials significantly impacts critical attributes of deposited films, including their quality, morphological characteristics, and crystalline structure.^14^ A number of reports have explored the influence of various precursor materials on the growth of solution-deposited ZnS thin films.^31−34^ Various zinc sources, such as zinc chloride, zinc acetate, and zinc nitrate, have been previously studied. The use of these zinc sources has been found to have noticeable effects on the ZnS films, leading to variations in film thickness and structural properties.^35−39^

A correlation has been reported between the crystalline quality and the optical and electrical characteristics of Zn-based films grown by metal–organic chemical vapor deposition on GaAs.^40^ This relationship was intricately tied to substrate oxidation, a phenomenon that mitigates the significance of Zn^2+^ ions’ absorption on the surface during chemical deposition.^40^ Notably, despite the mitigating effect on Zn^2+^ ions, certain quantities have been identified as positively impacting the prevention of air oxidation on the nascent GaAs surface.^41^

There is a notable gap in the existing literature regarding the formation of thin films of zinc chalcogenides from solutions, representing a critical lack of understanding of the intricate interplay at the surface–solution interface.^36−38,42−44^ It is noteworthy that different anions influence this interaction, an effect that has been somewhat overlooked but bears substantial importance for understanding the early stages of thin film growth. In this paper, we investigate a range of factors dictating the formation of ZnS films from solution, while highlighting the intricate effect of the different counteranions (Zn^2+^ salts) present in solution. This includes substrate surface wetting by the solution, adsorption of Zn^2+^ ions, substrate oxidation damage, formation of passivation layers, oxygen trapping, nucleation and growth of ZnS nanocrystallites, and the overall dynamics of film growth. Additionally, the study explores stress accumulation and subsequent fracture leading to crack formation within the film. We investigated the issue of adhesion and its correlation with the quality of solution-deposited ZnS films, elucidating the mechanism related to film cracking.^45−48^ By employing surface science methodologies, the present study expands our understanding of solution deposition of ZnS thin films, laying a foundation for the development of a straightforward, cost-effective technique for the deposition of ZnS thin films for a myriad of potential applications.

## Experimental Section

### Materials

Zn(CH_3_COO)_2_·2H_2_O (≥99.99%), ZnCl_2_ (≥99.99%), Zn(NO_3_)_2_·6H_2_O, ZnI_2_ (≥99.99%), ZnSO_4_·7H_2_O (≥99.99%), hydrazine hydrate (N_2_H_4_ 50–60%), NH_4_Cl (≥95%), and thiourea (≥99.0%) were purchased from Sigma-Aldrich and used without further purification. Ammonium hydroxide (25%w/w), acetone (Bio-Lab, technical grade), and 2-propanol (Bio-Lab, 99.8%) were used without further purification. Distilled water (DIW) was obtained from a Millipore Direct Q3 water purification system. Monocrystalline GaAs(100) wafers (undoped, epi polished with ±0.1 miscut) were manufactured by AXT Inc. and purchased from Geo Semiconductor (UK) Ltd.

### Substrate Preparation

GaAs (100) substrate wafers were cleaved into 1.5 × 2 cm^2^ rectangles and sonicated in Contrad 70 detergent solution (Decon Laboratories, PA) for 5 min at 40 °C. Wafers were then rinsed with DIW, acetone, and 2-propanol. Finally, the substrates were dried under a stream of pure N_2_ gas.

### Deposition Procedure

The aqueous deposition solutions each contained 0.2 M Zn^2+^ salts: Zn(CH_3_COO)_2_ (ZnAc_2_), ZnCl_2_, Zn(NO_3_)_2_, ZnI_2_, or ZnSO_4_. Other reagents added to the deposition bath were 7.56 M hydrazine hydrate, 1.3 M NH_4_OH, and 0.145 M NH_4_Cl. The final solution volume was 52 mL. The last reagent added was the sulfide anionic precursor thiourea (final concentration 0.2 M) and immediately afterward the GaAs substrates were immersed in the deposition solution. Depositions were carried out at 80 °C with a range of deposition times from 2 to 120 min. In addition, reference samples were prepared by treating substrates for 2 min at 80 °C in solutions containing the exact same composition described above while excluding the thiourea reagent. This was done to prevent ZnS film formation for the sake of evaluating the interaction between the solution and substrate surface.

## Characterization

### X-ray Diffraction (XRD)

XRD characterization was carried out using a Panalytical Empyrean Diffractometer equipped with a position sensitive X’Celerator Detector. X-ray wavelength was λ = 1.5405 Å (Cu Kα radiation). A 2θ range of 20–35° was scanned at 6.25° min^–1^ steps. Measurements were carried out with a sample offset angle of 2° to prevent masking of the signal originating from the thin films of interest by the intense peaks from the monocrystalline substrate.

### High-Resolution Scanning Electron Microscopy (HRSEM)

Using FEI Verios 460L HRSEM, images were obtained in plan-view and cross-sectional sample geometries. Beam currents of 25 to 50 pA and acceleration voltages ranging from 3 to 5 kV were used. HRSEM cross-sectional images revealed the thickness of the ZnS thin film, as shown in Figure S1. The film thickness was consistently measured from the cross-sectional images from all of the precursors studied. The film thickness was calculated as an average of three values obtained from each image, and the error bars were calculated from these data sets.

### X-ray Photoelectron Spectroscopy (XPS)

Measurements were conducted using an Escalab Xi spectrometer at a base pressure of 1 × 10^–9^ mbar. A monochromatic Al Kα X-ray source with an energy of 1486.6 eV was employed, along with a reference binding energy peak position of the C 1s core-level line at 284.8 eV. XPS signals for As 3d, Zn 2p, and Ga 3d core levels were collected to detect the oxidation states of the elements and chemical bonding peculiarities of the thin films and substrate. This was done for pristine surfaces cleaned for 180–200 s with gentle Ar^+^ ions etching using a cluster gun: primary acceleration energy (*U*_0_) −8 kV, Ar cluster size 150 au, beam current 0.01 mA, and scanning area 2.5 × 2.5 mm. The cleaning time was selected based on test profiling with 30 s step size and simultaneous detection of decreasing intensity of C 1s core-level lines arising from surface contaminants. Samples of GaAs with adsorbed Zn^2+^ cations were cleaned, exposed to vacuum, and moved to the XPS preparation chamber. This procedure prevented the formation of natural oxides on the GaAs substrate surface. XPS analysis was performed for 5 samples after adventitious carbon removal during 180 s with a cluster Ar^+^ gun (*U*_0_ = 4 kV, cluster size 500 au, and etching area 2.5 × 2.5 mm). Spectra were normalized to the total signal obtained for each sample using the following equation: × 10^6^, where *i* is the number of XPS energy channels, *k* is the sample number, and  is the measured intensity in the *i*_th_ channel for the *k*_th_ sample.

### Transmission Electron Microscopy (TEM)

Thin (<100 nm) lamellae were extracted under the standard lift-out procedure using a Thermo Fisher Helios G4 dual-beam tool. The final thinning was carried out with a primary ion energy of 16 kV to avoid amorphization of the ZnS layer.

TEM and high-resolution lattice imaging (HRTEM) analyses were carried out on a probe-corrected Spectra 200 TEM operating at 200 kV. EDS mapping was carried out with in-column 4-fold EDS detectors that enabled the unveiling of low-intensity peaks and the reduction of exposure time of the specimens to the high-energy electron beam, thus minimizing electron beam radiation damage.

## Results and Discussion

**Figure 1:**
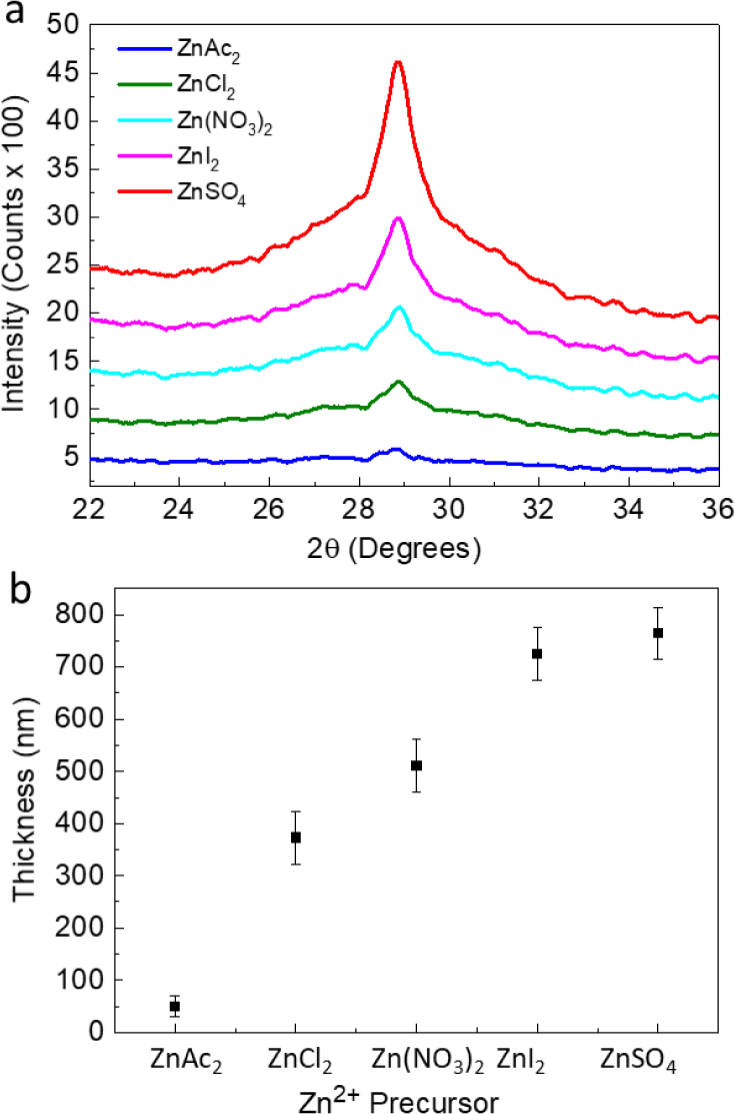
ZnS thin films deposited from solution onto GaAs (100) substrates for 2 h at 80 °C using different Zn^2+^ precursors. (a) X-ray diffractogram (b) thickness, obtained from HRSEM of cross-section samples.

In the first stage of the research, ZnS thin films were deposited from solution on GaAs (100) using various Zn^2+^ precursors under experimental conditions previously shown to give ZnS thin films.^46^Figure 1 shows XRD analysis for films deposited with Zn(CH_3_COO)_2_ (denoted henceforth as ZnAc_2_), Zn(NO_3_)_2_, ZnCl_2_, ZnI_2_, and ZnSO_4_ precursors for 2 h at 80 °C. The XRD pattern of ZnS thin films deposited onto the GaAs (100) substrate (Figure 1a) showed a single Bragg reflection corresponding to the (002) peak of wurtzite ZnS or the (111) peak of zincblende ZnS. The different results obtained with different zinc salts confirmed that the zinc source indeed has a pronounced effect on the resulting films.

For the acetate, chloride, and nitrate precursors, a weak peak is discernible, indicating a diminutive thickness or noncrystalline nature of the films. In contrast, the iodide and sulfate precursors exhibit a noticeably stronger Bragg peak, suggesting a higher growth rate or a comparatively larger crystalline component relative to layers produced with the aforementioned precursors. However, it is demonstrated that the diffraction pattern associated with the sulfate (SO_4_) species clearly reveals an “amorphous mound” at 2θ values between 25 and 32 degrees. This observation points out the presence of a substantial amorphous constituent within the films alongside the crystalline structural component.

The thickness of the films prepared with different precursors was evaluated from cross-sectional HRSEM images (Figure 1b). It is evident that the film thickness is strongly dependent on the Zn^2+^ precursor used, with the lowest deposition rate obtained for the acetate source, while the highest deposition rate was obtained for the case of sulfate.

**Figure 2:**
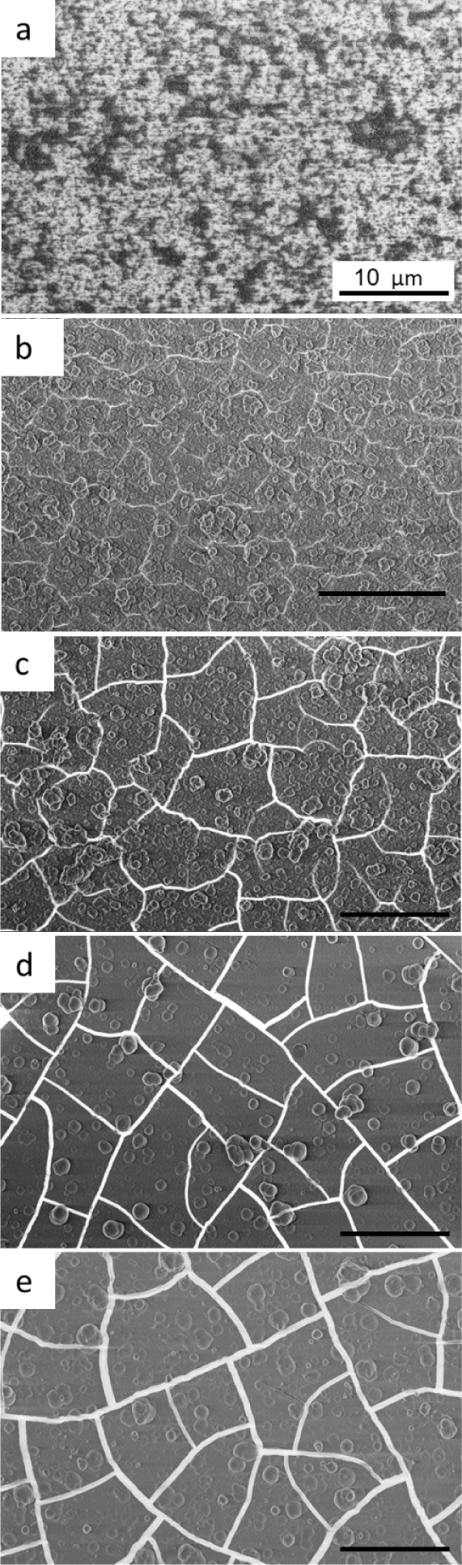
HRSEM plan-view images for ZnS thin films deposited onto GaAs (100) substrates for 2 h at 80 °C, using different zinc precursors: (a) Zn(Ac)_2_, (b) Zn(NO_3_)_2_, (c) ZnCl_2_, (d) ZnI_2_, and (e) ZnSO_4_. All scale bars correspond to 10 μm.

Additionally, plan-view HRSEM images in Figure 2 show the morphology for films deposited using various precursors for 2 h at 80 °C. The films formed from the acetate salt are characterized by small (75 ± 10 nm) clusters (Figure 2a) and exhibit no cracks. By contrast, the remaining films (Figures 2b–e) appear to be continuous yet cracked, and rounded clusters are seen on the surface of the films. The size of the clusters and the crack openings increase in the following order of precursors: ZnAc_2_ < Zn(NO_3_)_2_ < ZnCl_2_ < ZnI_2_ < ZnSO_4_.

The plot shows a remarkable evolution in the cracking pattern, characterized by a gradual reduction in complexity as the intricate network of fine cracks with curved shapes, and branching gradually transforms into a simpler and more straightforward arrangement of thicker cracks. Such a transition in the cracking pattern not only offers valuable insights into the underlying mechanics of crack propagation but also underscores the dynamic nature of material behavior under external forces. For example, this indirectly depicts the accumulation of high residual stresses in the continuous film,^49^ which can cause cracking under shear, impact, or compressive external loads that can take place during further photovoltaic device manufacturing steps and exploitation.

Though previous studies have reported the problem of cracking in solution-deposited ZnS thin films, they did not venture to offer explanations or solutions to this problem.^24,27^

**Figure 3:**
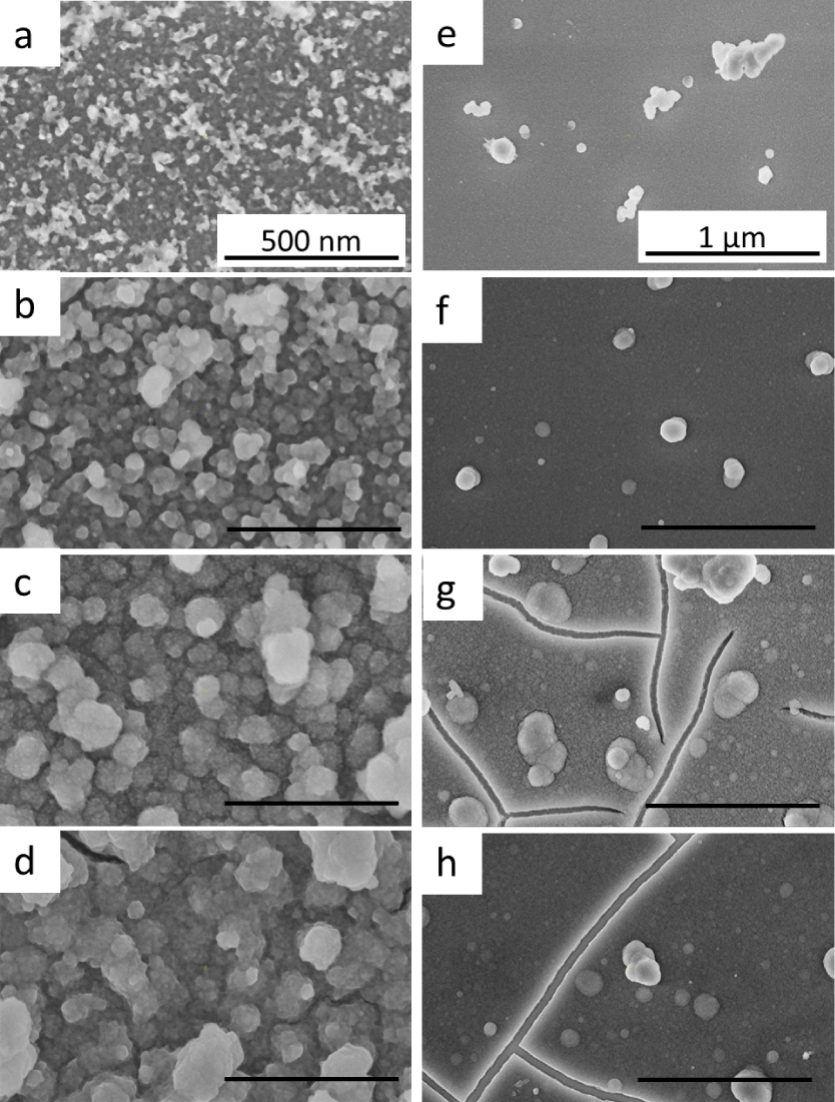
HRSEM plan-view images of ZnS thin films deposited onto GaAs (100) substrates at 80 °C for 2, 5, 10, and 15 min, respectively. Zn^2+^ precursors used were (a–d) ZnCl_2_ and (e–h) ZnSO_4_. Scale bars in (a–d) correspond to 500 nm, while scale bars in (e–h) correspond to 1 μm.

Based on the above results, two distinct precursors, ZnSO_4_ and ZnCl_2_, were selected for studying the early stages of ZnS film growth. Deposition experiments were carried out on GaAs (100) substrates for brief intervals of 2, 5, 10, and 15 min. The HRSEM micrographs of the obtained films are presented in Figure 3.

According to the results shown in Figures 3a–d, a direct correlation appears between the deposition time and grain growth, as expected from the classical theory of nucleation and growth.^14^ Notably, it is evident that during the initial 15 min deposition period, no cracks are observed in films deposited from the ZnCl_2_ precursor. Consequently, this observation implies that the crack formation process in this deposition scenario is relieved by intensive formation of grain boundaries. This may also result in some degree of inhibition of film growth, which in the case of the ZnCl_2_ precursor manifests itself in a lower average growth rate relative to films grown using alternative precursors. On the other hand, the progressive evolution of cracking in films deposited from the ZnSO_4_ precursor is initiated already between 5 and 10 min of growth as shown in Figure 3f,g. Notably, they exhibit continuity and uniformity as early as 2 min into the deposition process.

**Figure 4:**
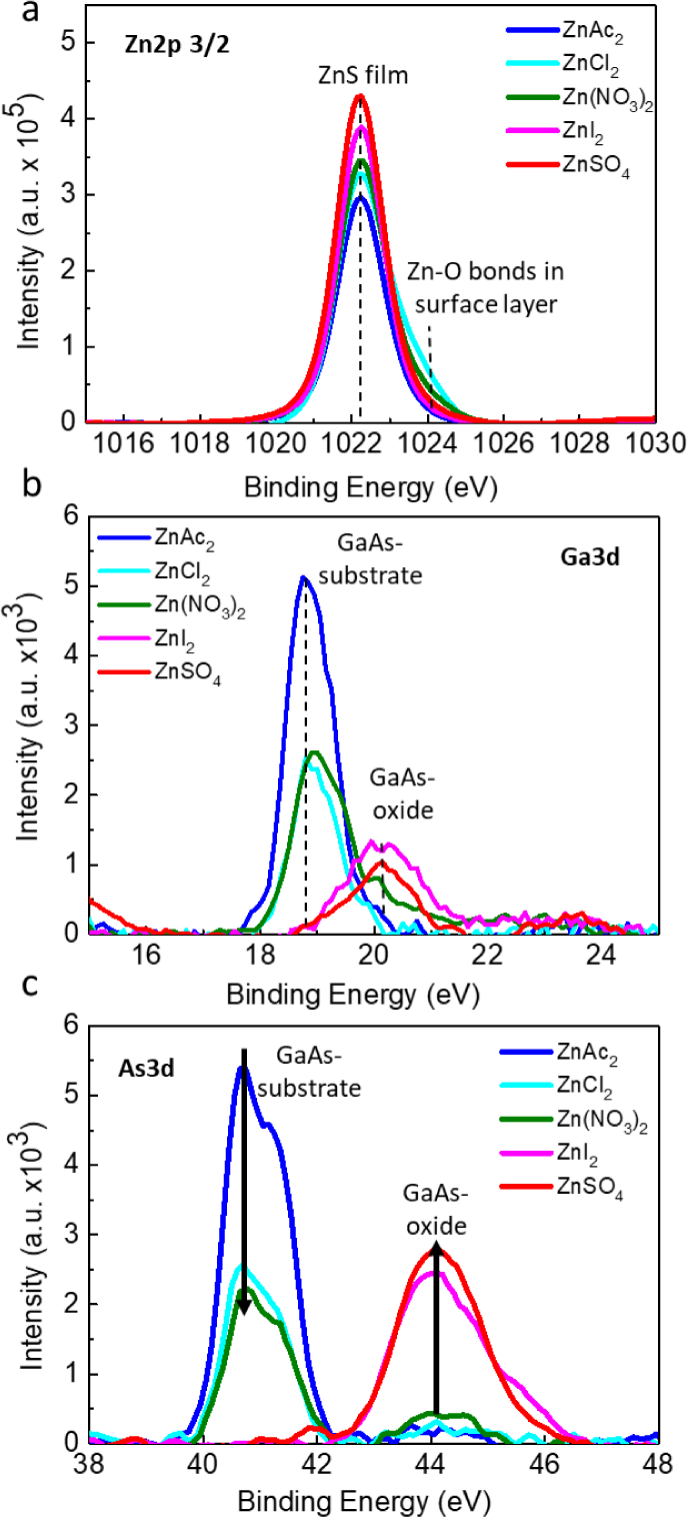
XPS analysis of the surface composition and substrate oxidation for ZnS films deposited for 2 min. (a) Zn 2p 3/2, (b) Ga 3d, and (c) As 3d XPS core-level spectra. Spectra are normalized to the total signal obtained for each sample.

To investigate the effect of different precursors on the early stages of growth, XPS characterization was carried out on ultrathin films deposited for a short duration of 2 min. Figure 4 shows the XPS results obtained for the films deposited for 2 min from the 5 types of Zn precursor solutions. The Zn 2p3/2 core-level XPS line intensity in Figure 4a indicates that the concentration of Zn^2+^ cations on the surface varies with the precursor type according to CH_3_COO ^→^ NO_3_ → Cl^–^ → I^–^ → SO_4_^–2^. The peak position at ∼1022.2 eV corresponds to Zn bound to sulfide in nanocrystalline ZnS,^50,51^ slightly shifted (0.3 eV–0.5 eV) toward higher binding energies. For the NO_3_^–^ and the Cl^–^ precursors, after 2 min of deposition, high-energy tails were observed for the Zn 2p3/2 core-level lines. This shoulder peak is observed at a binding energy (BE) of ∼1023.5 eV, which is significantly higher (by ∼1.5 eV) than that of ZnO expected at 1022.0 eV, ruling out the possibility of ZnO formation. These shifts of Zn–S and Zn–O components toward higher binding energies are well-explained in the literature as a confinement effect which occurs in thin films or nanocrystalline materials on the observed core-level binding energies.^52^ The observed shift can reach values greater than 2 eV for ultrathin ZnO films under 10 nm thickness and is related to exciton confinement phenomena in nanostructured semiconductors. Thus, we can conclude that we are observing a transition state from an ultrathin interfacial wetting layer strongly interacting with the GaAs surface to a continuous, fully coalesced, and thicker ZnS film. Thus, the high-energy shoulder corresponds to an ultrathin oxidized layer, and the low-energy peak in the Zn 2p3/2 XPS spectra corresponds to ZnS nanocrystal nuclei formed at early stages of deposition from Zn(NO_3_)_2_ and ZnCl_2_ solutions. This is also confirmed by the STEM-EDS results shown later in Figures 6 and 8. This phenomenon was not observed for the ZnAc_2_ precursor due to the absence of oxidation of either the film or substrate and formation of an ultrathin ZnS wetting layer during the deposition process. Additional support for this claim comes from the very high intensity of Ga and As peaks (strong signal from the substrate due to weak attenuation by the ultrathin film) observed in this sample (Figure 4b,c).

The 2 peaks of Ga 3d and As 3d in Figure 4b,c, respectively, correspond to (i) the spin–orbit split (3d5/2 and 3d3/2) signal from the clean GaAs substrate beneath the ZnS film (binding energies Ga 3d5/2 = 18.8 eV and As 3d5/2 = 40.7 eV, correspondingly) and reflect the formation of an ultrathin film of thickness less than 3 nm (according to the inelastic mean free path estimation for electrons λ ) or a discontinuous film with exposed areas of the GaAs substrate (see also SEM in Figures 2 and 3); (ii) the broad peaks around 20.0 and 44.0 eV for Ga 3d and As 3d lines, respectively, reflect oxidation of GaAs. The oxide peak in the Ga 3d and As 3d spectra shows that during ZnS deposition from ZnSO_4_ and ZnI_2_ precursor solutions, notable oxidation of the GaAs surface occurs. On the other hand, for ZnAc_2_ and Zn(NO_3_)_2_, only weak oxidation of the surface was found after 2 min of deposition. The ZnCl_2_ precursor tends to form O–Me–S bonds near the GaAs surface, characterized by lower BE as compared to As–O in cases of ZnSO_4_ and ZnI_2._ This bond has higher As involvement than Ga, as seen from the contrast of the lower relative intensity of the oxide peak in the Ga 3d spectra (Figure 4b) compared to that in the As 3d spectra (Figure 4c), which is an order of magnitude smaller than that obtained for films grown from ZnI_2_ and ZnSO_4_-based solutions.

**Figure 5:**
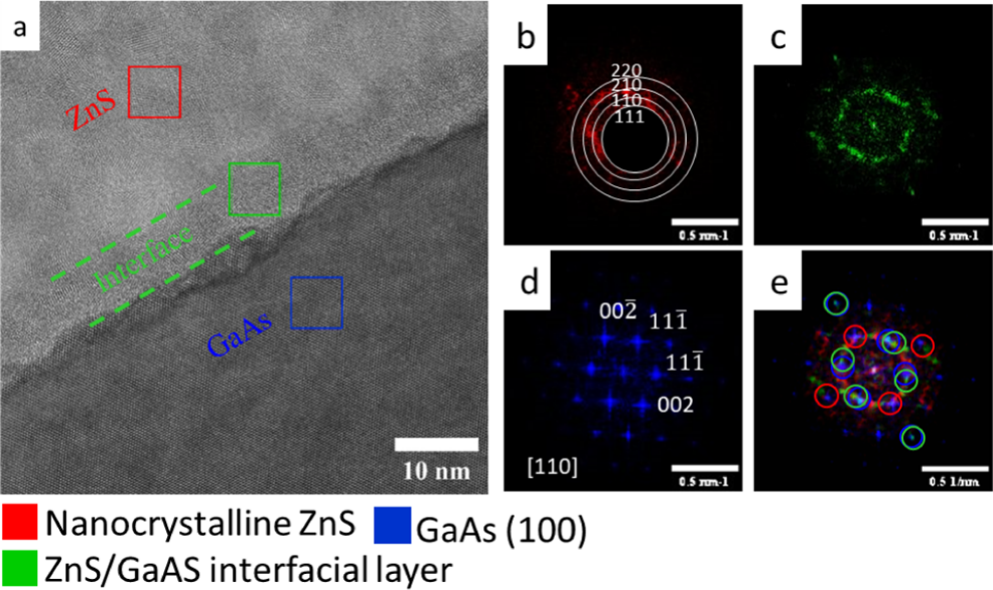
(a) HRTEM of the ZnS/GaAs interface obtained from a cross-sectional specimen prepared from a ZnS film after 5 min of deposition from a ZnSO_4_ precursor bath. FFT analysis, demonstrating the presence of (b) red, the ZnS film; (c) green, an intermediate layer between the ZnS and the GaAs substrate; and (d) blue, GaAs(100) substrate. (e) An overlay of the data in (b–d) showing the orientation relations between the layers.

The HRTEM analysis of the microstructure of a ZnS film thin cross-section, lifted-out by SEM/FIB from the sample grown from a ZnSO_4_ precursor solution for 5 min, is shown in Figure 5a. The ZnS film is nanocrystalline with a crystallite size of 5–10 nm. The ZnS layer is grown on top of a 10–15 nm thick nonuniform interfacial layer that appears to form on the damaged GaAs surface. FFT analysis of three areas in the HRTEM image color-coded in red, green, and blue pointed out the presence of a ZnS film, an interfacial layer, and the GaAs substrate, as shown in Figures 5b–d, respectively.

**Figure 6:**
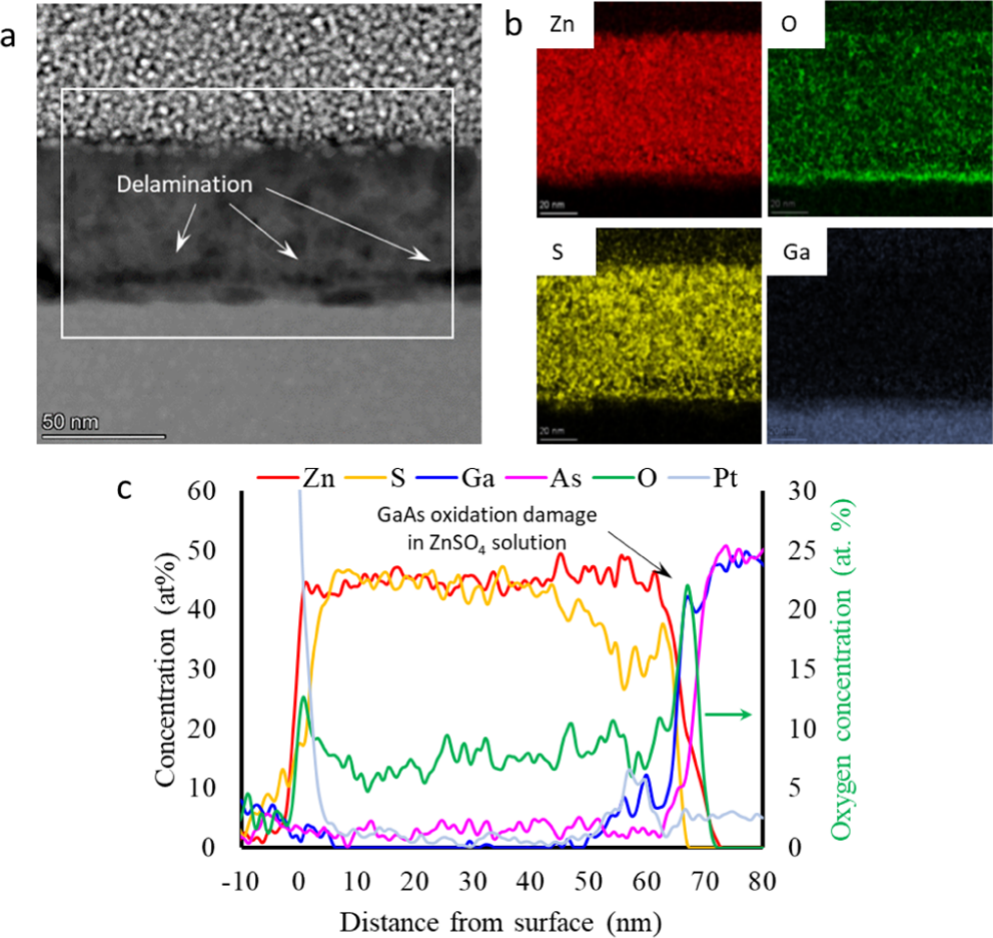
Elemental composition mapping carried out in the TEM for ZnS films deposited for 5 min from a ZnSO_4_ precursor bath. (a) STEM image and (b) corresponding EDS maps for zinc, sulfur, oxygen, and gallium. (c) EDS line profiles of the different elements along the cross-section of the sample.

STEM/EDS analysis of the elemental distribution over the cross-section of the film, shown in Figure 6, revealed the sharp arsenic- and oxygen-rich layers formed on the GaAs surface. From the STEM image in Figure 6a, as well as from the elemental maps of zinc, sulfur, oxygen, and gallium shown in Figure 6b, it is evident that the interfacial layer tends to delaminate from the 50–55 nm thick ZnS film under the electron beam irradiation (see also STEM data in Supporting Information, Figure S3), leaving a 5–10 nm thick oxidized intermediate layer on the substrate. The intermediate layer, where delamination occurs, shows a lower sulfur concentration as compared to the ZnS layer according to the line profile data extracted from elemental maps (Figure 6c), which may weaken the bonding strength between the ZnS and underlying surface due to the formation of a defective, vacancy-rich metastable structure. This irradiation damage can aid in modeling the behavior of the interface under stress accumulation during film growth, as discussed in the last section of this manuscript.

**Figure 7:**
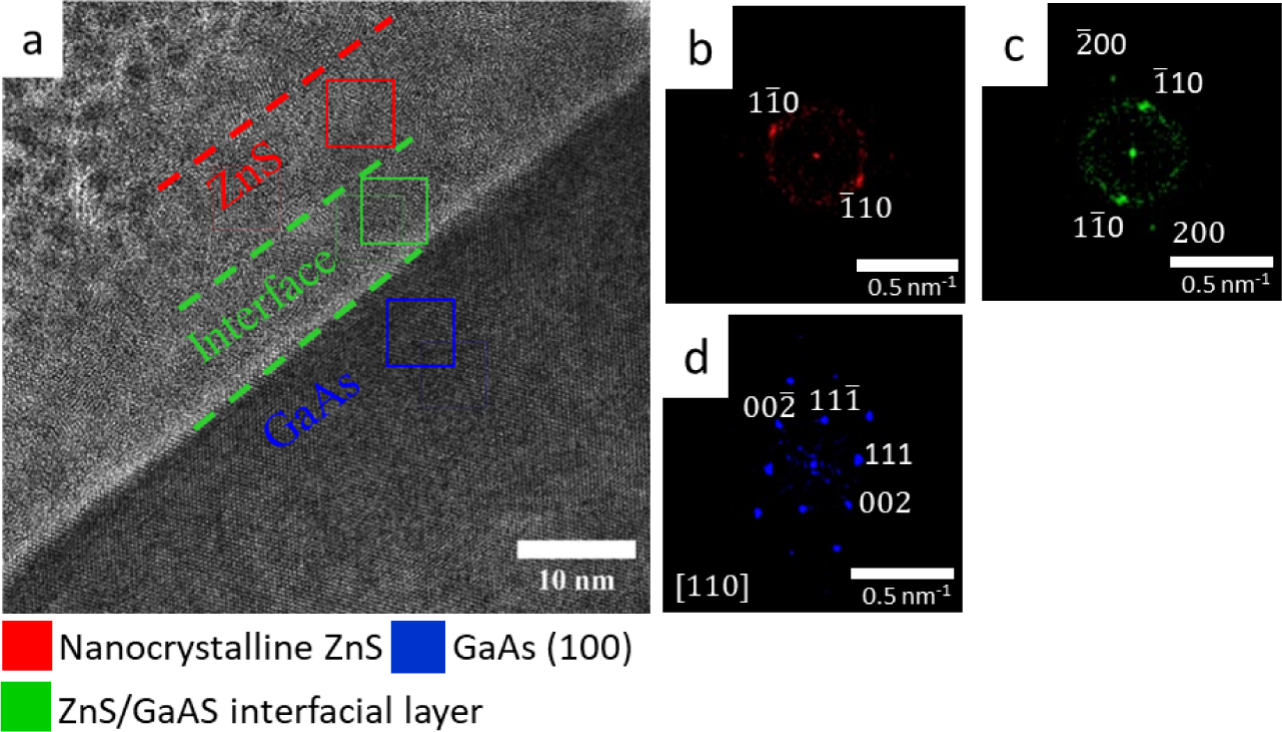
(a) HRTEM of the ZnS/GaAs interface obtained from a cross-sectional specimen prepared from a ZnS film deposited for 5 min in a ZnCl_2_ precursor bath. FFT analysis, demonstrating the presence of (b) red, the ZnS film; (c) green, an intermediate layer between the ZnS and the GaAs substrate; and (d) blue, the GaAs(100) substrate.

HRTEM analysis of the cross-sectional ZnS film specimen, grown for 5 min from ZnCl_2_-based solution, is shown in Figure 7. The ZnS layer at the early stages of film formation in this solution has a relatively lower thickness of 20–40 nm with a developed surface, demonstrating the growth of individual crystalline nuclei, as seen in Figure 7a. Figures 7b–d shows FFT patterns obtained from the ZnS film (red), the interfacial layer (green), and the GaAs substrate (blue), respectively. In contrast to the case of growth from the ZnSO_4_ precursor (compared with Figure 5a), the interface layer is somewhat thicker, and the underlying GaAs surface is extremely flat, showing no oxidation damage.

**Figure 8:**
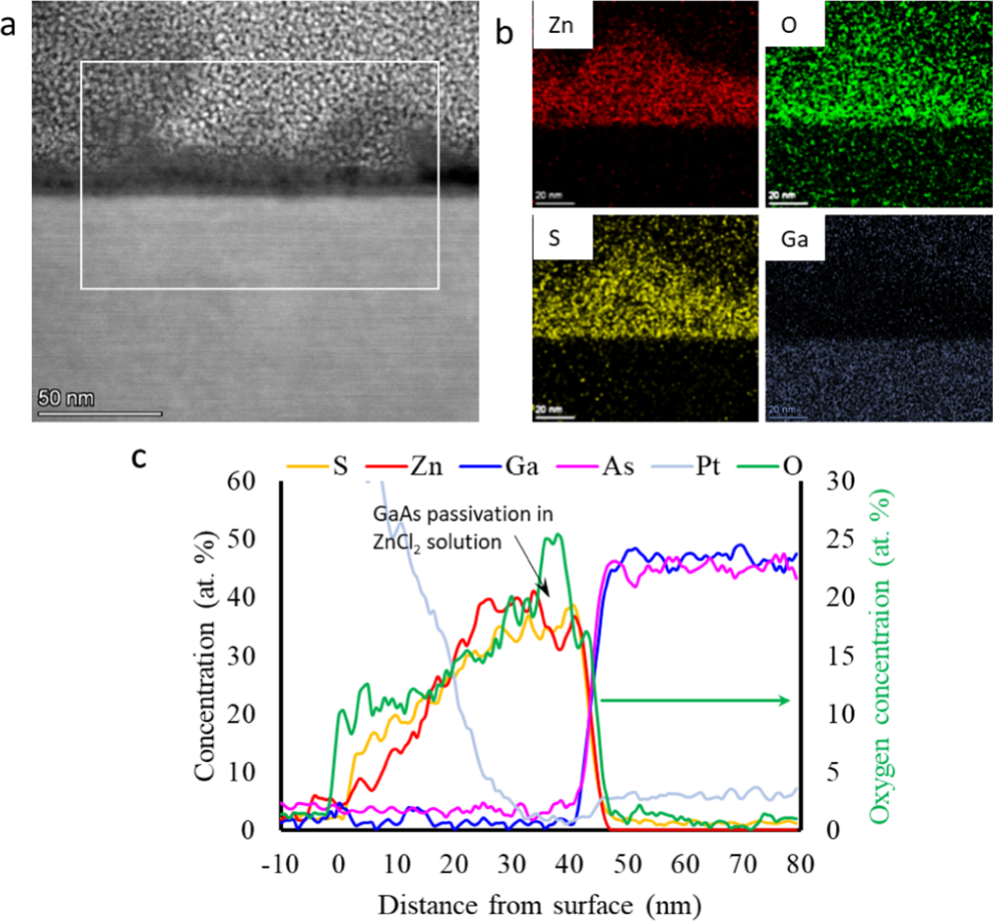
Elemental composition mapping for ZnS films deposited for 5 min from a ZnCl_2_ precursor bath: (a) STEM image; (b) corresponding EDS maps for zinc, sulfur, oxygen, and gallium; (c) EDS line profiles of the different elements along the cross-section of the sample.

The STEM/EDS analysis of the elemental composition distribution shown in Figure 8 allowed us to understand the early stages of ZnS growth from ZnCl_2_ solution in the absence of surface oxidation. It was found that oxygen is trapped in the ZnS layer with some enrichment near the interface, leading to preservation of the surface from damage and improvement of film adhesion.

Comparison of the HRTEM and EDS data of the 2 samples in Figures 6 and 8 points to strong GaAs surface oxidation damage in the case of growth from the ZnSO_4_ precursor. The interfacial 3–5 nm of GaAs has a very high concentration of oxygen, and the Ga:As 1:1 ratio is broken. This result is in line with the XPS analysis shown in Figures 4b,c. Moreover, STEM imaging revealed a weak adhesion of ZnS to the GaAs oxide, causing further delamination of the layer in the interface region, as seen in Figure 6a.

At the same time, ZnS films grown from the ZnCl_2_ solution clearly have a nonuniform surface, and nucleation still plays an important role after 5 min of deposition. The interface morphology does not exhibit substrate surface damage, implying that the ZnS films have better adherence to the substrate and accumulate less stress at the early stages of deposition.

There is also the important observation of oxygen trapping by the primary nuclei in the case of ZnS grown from a ZnCl_2_ solution. The oxygen concentration gradually decreases from the substrate to the surface, allowing the formation of ZnS with a nearly 1:1 stoichiometry, with an abrupt reduction in S concentration and a corresponding sharp rise in O toward the interface with the substrate.

Notably, TEM analysis shows that delamination of the film (see Figure S2) along with the consequent brittle cracking takes place together with strong oxidation of the surface depending on the precursor solution type (see contact angle results in Figure S3).

**Figure 9:**
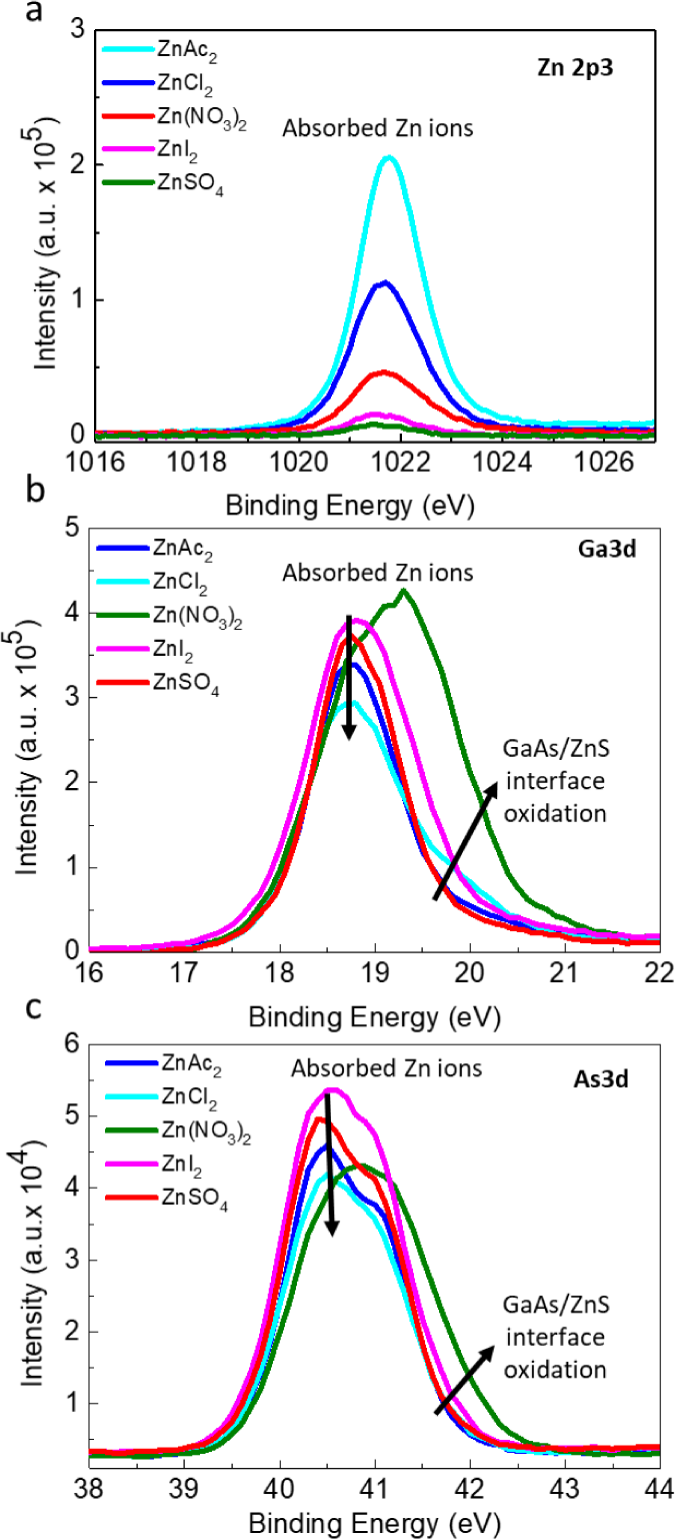
XPS of GaAs substrates following exposure to solutions containing different Zn^2+^ precursors. (a) Zn 2p3/2; (b) Ga 3d; (c) As 3d. Spectra are normalized to the total signal obtained for each sample.

From the XPS and TEM analyses in Figures 4–8, we can also conclude that nucleation and growth of the ZnS films strongly depend not only on the precursor type itself but also on the interaction of the solution with the GaAs surface. Due to the critical role of the interaction of the counteranions of the Zn^2+^ salt precursors and the GaAs surface, samples of GaAs from the same batch were exposed to a solution containing 0.2 M Zn(CH_3_COO)_2_/ZnCl_2_/Zn(NO_3_)_2_/ZnI_2_/ZnSO_4_, 7.56 M hydrazine hydrate, 1.3 M NH_3_, and 0.145 M NH_4_Cl with a final solution volume of 52 mL. Namely, the substrates were exposed to the deposition solution, while thiourea (sulfide precursor) was omitted from the bath to prevent ZnS formation. The surfaces of the resulting 5 samples were studied with XPS, and the results are shown in Figure 9.

The Cl^–^ and I^–^ precursor solutions exhibit the strongest surface passivation, resulting in oxidation of the GaAs surface, as manifested by the shift of the Ga 3d and As 3d peaks toward higher binding energies as compared to the solutions with nonhalogenide, oxygen-containing anions.

**Figure 10:**
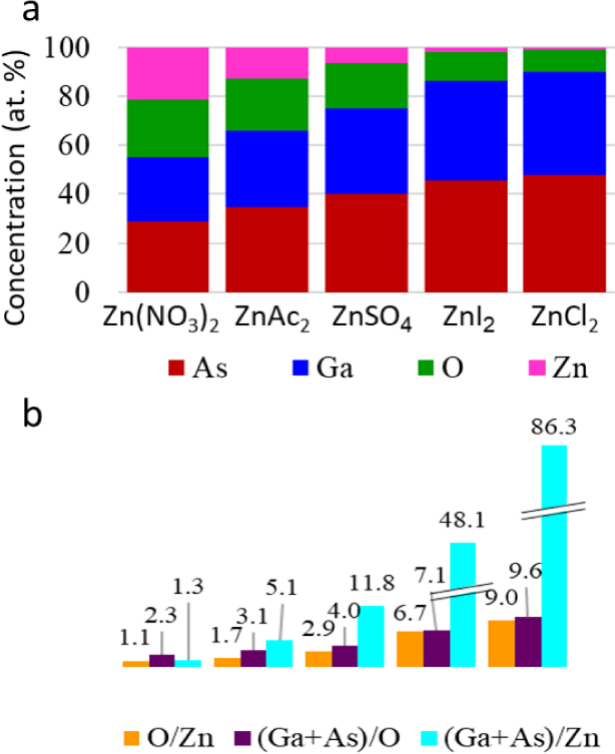
Quantitative analysis of the XPS data in Figure 9. (a) Surface composition and (b) surface coverage rate by zinc and oxygen (right) after GaAs substrate treatment in various Zn-containing solutions.

The alteration in surface composition was further quantified by the XPS data. Figure 10 illustrates how in the absence of S^2–^ anions, Zn^2+^ cation adsorption on GaAs increases with decreasing oxidation of GaAs, as mainly seen from Ga 3d and As 3d peaks at BE near 18.5 and 40.5 eV, respectively, reflecting the fact that no strong oxidation of the surface is present (for example, Figure 4 with oxide BE for As 3d and Ga 3d around 20.0 and 44.0 eV, respectively). The adsorption rate of Zn^2+^ cations sharply decreases due to the formation of the passivation layer on the GaAs surface, as is clearly seen from comparing the XPS analysis data from Figures 9 and 10 for ZnCl_2_- treated substrate, where the highest O/Zn ratio was found accompanied by minimal absorption of Zn^+^ cations. We note that no evidence was obtained for the formation of a substantial oxide layer following the exposure of the substrates to solutions of the various Zn^2+^ precursors. XPS survey spectra in Figure S4 indicated the presence of oxygen mostly from adsorbed oxygen-containing counteranions, sulfate and nitrate.

The XPS analysis indicates that surface modification via Zn^2+^ cation adsorption takes place in different manners for different anions. The amount of Zn^2+^ adsorbed onto the GaAs substrate, as reflected from the Zn:GaAs XPS peak ratio, varies from approximately 1:1 for Zn(NO_3_)_2_ to 1:86 for the ZnCl_2_ solution (see Figure 10). A clear inverse correlation was found between the intensity of the Zn 2p XPS line and that of high binding energy components (indicating oxidation) in the As 3d and Ga 3d XPS spectra. This correlation can be explained by the surface passivating effect of the solutions, which inhibits adsorption of Zn^2+^ cations on the GaAs surface.

It is well-known that film quality and crack formation are strongly dependent on surface wetting by the solution.^46^ To analyze the change in wetting of the substrate following treatment with deposition solutions containing different anionic sources for Zn^2+^, the water–substrate contact angle Θ was measured after treating the GaAs substrates for 2 min in the above solutions, while excluding the sulfide precursor to prevent ZnS film formation. This experimental protocol models the early stages of nucleation and sheds light on the chemical modification of the substrate surface during the early stages of ZnS film deposition.

During the wetting process, the change in free energy is expressed by eq 1:^53,54^
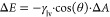
1

where Δ*A* is the solid–vapor surface area, θ is the contact angle, and γ_lv_ is the free energy of the liquid–vapor interface. For a hydrophilic surface, the contact angle θ is <90° which means that the free energy change of wetting is negative; i.e., the wetting of the surface occurs spontaneously.

Measurement of the contact angle allows one to qualitatively estimate the substrate surface modification caused by the interaction between the corresponding solutions and the substrate surface.

**Figure 11:**
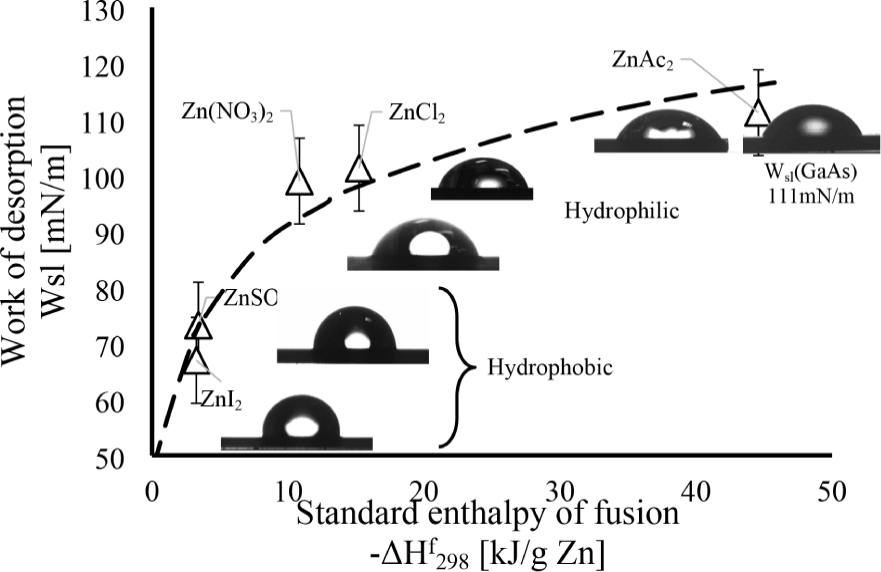
Evolution of GaAs surface wettability for treatments with different Zn^2+^ cation precursors, represented by the enthalpy of fusion of the various Zn^2+^ salts.

The analysis in Figure 11 shows a clear dependence of the surface contact angle on the cation present in solution: AC > Cl ^–^> NO_3_^–^ > SO_4_^2–^> I^–.^ This confirms the importance of concurrence between substrate passivation and oxidation damage during the interaction with the various solutions, as shown also by XPS analysis. The low deposition rate and ultrathin ZnS film formation, as well as oxygen trapping by the films, lead to the formation of passivated surfaces and, as a result, improved adhesion with low tendency for delamination. Delamination, in turn, causes the partial release of stress, but, due to the continuation of film growth (especially with a high growth rate such as in the use of ZnSO_4_ or ZnI_2_ precursors), the film itself continues to further accumulate stress. Consequently, it leads to cracking by the mechanism described below.

Crack propagation is arrested when the stress is below a critical value as given by eq 2 here:^55^
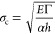
2

where *E* is the bulk elastic energy stored in the film and Γ is the surface energy per unit of the crack surface area which is higher for a hydrophilic surface; thereby, less energy will be required to initiate crack formation. The α parameter depends on the relative elastic moduli and adhesion of the film to the substrate. Assuming that the film has a much lower modulus than the substrate, α = 0.5 if the film is bound on both sides and α = 1.25 if it is bound on one side only, where *h* is the film thickness.^56^

Peeling of the film from the substrate due to formation of the bond-weakening adhesion layer as shown by STEM (see Figure 6) can lead to cracking.^57,58^ The critical unbonded length for buckling *l*_c_ /*h* ∼ (*E*_f_/σ)1/2 (*h* is the film thickness, *E*_f_ is Young’s modulus, and σ is the stress accumulated in the film). *E*_f_ for the ZnS thin film is reported to be around 75 GPa,^59^ the documented modulus of rupture (bending strength) for bulk ZnS is 103.4 MPa, and the tensile strength reported in the literature for ZnS nanowires is 145–370 MPa.^60^ From this assumption, the *E*_f_/σ ratio can be estimated to be of the order 10^2^–10^3^, giving a critical length of buckling to thickness ratio *l*_c_/*h* ≈ 10–30. Thus, in the case of aggressive surface oxidation by the Zn^2+^ precursors or very long deposition time, having on the one hand large zones with delaminated film (high *l*_c_ values) and on the other hand continuously growing film, increasing the curvature of the surface, one can expect that accumulated stress will be relaxed by cracking of the material. The distance between individual cracks will be dependent on *l*_c_ and the kinetics of film growth. This explanation is clearly evidenced by SEM (Figures 2 and 3), where for the less aggressive precursors and lower growing rates, a very developed structure of the cracks was observed, with no serious damage to the thin ZnS film. On the other hand, aggressive passivation by the ZnSO_4_ and ZnI_2_ solutions leads to substrate oxidation and decreased wettability, rendering the surfaces almost hydrophobic.

It was also found that the bonding strength of the compound, represented by the enthalpy of fusion (standard enthalpy of formation −Δ*H*_f_^298^, normalized to the Zn mass in the solution, kJ/g), correlates well with the contact angle (see Figure 11) and work of desorption, as expressed by the Young–Dupré equation:
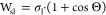
3

where *W*_sl_ is the work of desorption [mN/m], σ_l_ is the liquid surface energy (72 mN/m for water), and Θ is the contact angle, in degrees.

The values of standard enthalpy of formation used for the calculations taken from reference^61^ are shown in Table S1.

From the analysis of the surface condition of the GaAs substrate following treatment with various solutions of Zn precursors, we can conclude that the oxidation (dehydrophilization) of the surface and subsequent lack of adhesion and cracking can be explained by the formation of an interfacial layer with a tendency to delaminate following contact of the substrate with the solution. This hydrophobic layer formation, accompanied by Zn^2+^ adsorption, strongly depends on the precursor type, as is seen from XPS and contact angle analyses in Figures 9 and 11, respectively.

**Figure 12:**
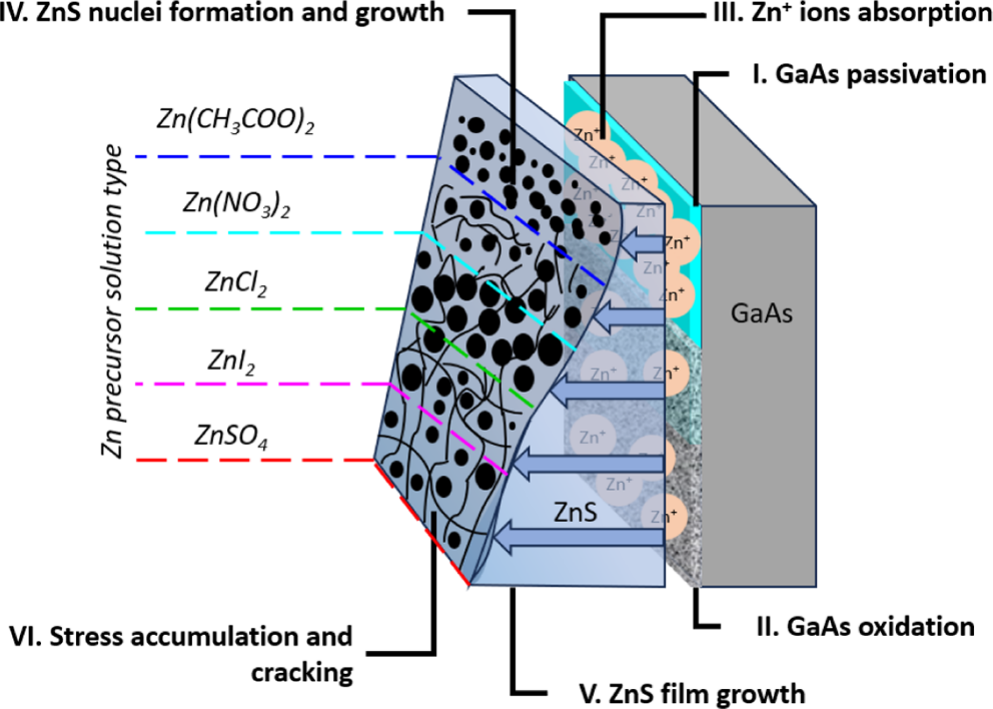
Graphical illustration summarizing the effect of the counterions on the growth rate and film microstructure.

After 5 min of deposition, the formation of the thin ZnS film and the wettability are similar for samples obtained from all the precursors (the contact angle is in the range of 74–79 deg) as presented in Figure S3. The sample deposited with ZnAc_2_ precursor with a very limited growth rate did not develop as a continuous film, reflecting a transition state between hydrophobic ZnS and bare GaAs with a measured contact angle of 86 degrees. A graphical illustration summarizing the insights gained in this research is presented in Figure 12.

## Conclusions

We presented a detailed study of the mechanism of ZnS thin film formation via chemical deposition from different precursors. It was shown that the early stages of film deposition play a key role in the quality of the ZnS films. The surface/solution interactions during the initial stages of deposition from the solutions containing Cl^–^, I^–^, and SO_4_^2–^ anions lead to the formation of a hydrophobic layer on the GaAs surface, preventing adsorption of Zn^2+^ from the solution. The weaker adhesion to the substrate was shown to stem from the presence of an interface layer with poor wettability, which was detected by XPS and TEM after 5 min of treatment of the substrate in the corresponding solutions. The subsequent deposition of the films results in cracking due to fast layer growth and accumulation of stress that is relaxed in the form of cracks in the absence of supporting media underneath.

In summary, for applications where the deposition time plays a dominant role, the usage of ZnSO_4_ or ZnI_2_ should be considered as a precursor for rapid (<2 min) deposition of a continuous ZnS wetting layer. In cases where the substrate surface needs to be maintained in its native state, the ZnAc_2_ precursor is preferred, while the deposition time should be significantly increased due to the slow growth rate. Due to the dominant nucleation process in solution in close proximity to the substrate when using the ZnCl_2_ precursor as a source of zinc ions, it is possible to obtain films up to 0.5 μm thick after 2 h of chemical deposition in a bath. In this case, cracking of the film occurs no earlier than 15 min after the start of the deposition process. Thus, we showed that for further development and scaling-up of the technology, the selection of the deposition parameters and precursors for chemical solution deposition of ZnS films should be based on the understanding of the solution–substrate interactions.

## Supporting Information Available

Additional data include standard enthalpy of formation used in Figure 11 for the various Zn^2+^ salts used in this work (Table S1), STEM images of cross-sectional specimen extracted from ZnS film (Figure S2), contact angle measurements for ZnS films (Figure S3), and XPS survey spectra (Figure S4) (PDF)
