# Supplementary material for: The Role of Counteranions in Solution Deposition of ZnS Thin Films on GaAs
Source: Inorg Chem. 2024 Dec 11;63(51):24104–14. doi: 10.1021/acs.inorgchem.4c03424 (PMC11684027; doi:10.1021/acs.inorgchem.4c03424)
Supplement: Supplementary file 1 — ic4c03424_si_001.pdf [file ic4c03424_si_001.pdf]

Supporting Information

The role of counter anions in solution deposition of ZnS thin films on GaAs

Noy Zakay[a,b], Shlomo Rand[a,b], Alexander Rashkovskiy[a,b], Nitzan Maman[b], Vladimir Ezersky[b] and Yuval Golan[ab]*

(a) Department of Materials Engineering, Ben-Gurion University of the Negev, Beer-Sheva 8410501, Israel

(b) Ilse Katz Institute for Nanoscale Science and Technology, Ben-Gurion University of the Negev, Beer-Sheva 8410501, Israel

*Corresponding author: ygolan@bgu.ac.il

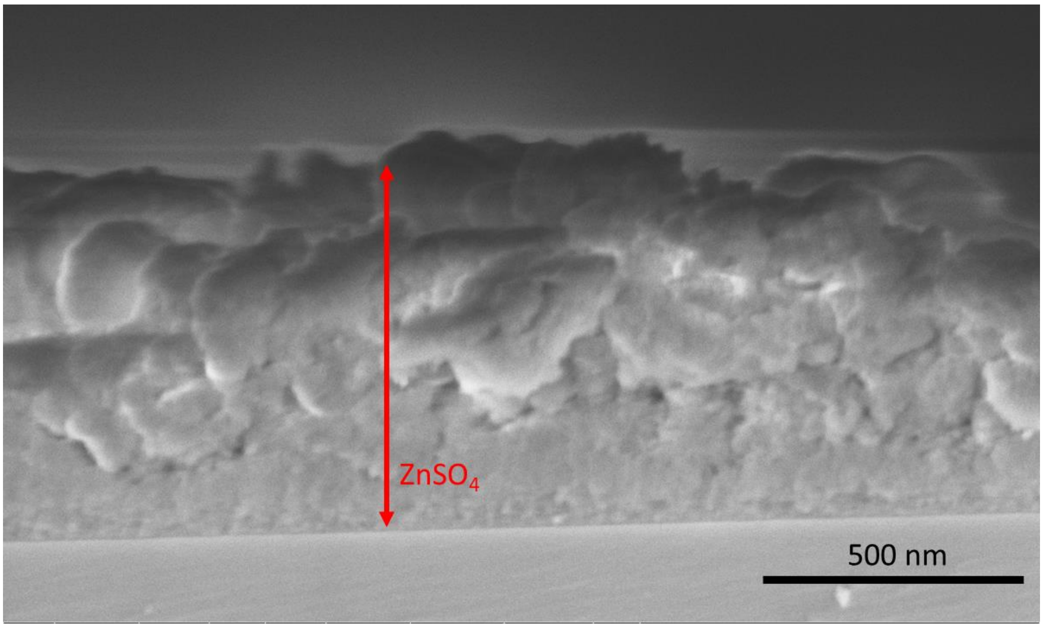

Figure S1. HRSEM cross-sectional image of the ZnS film deposited from $ZnSO_4$.

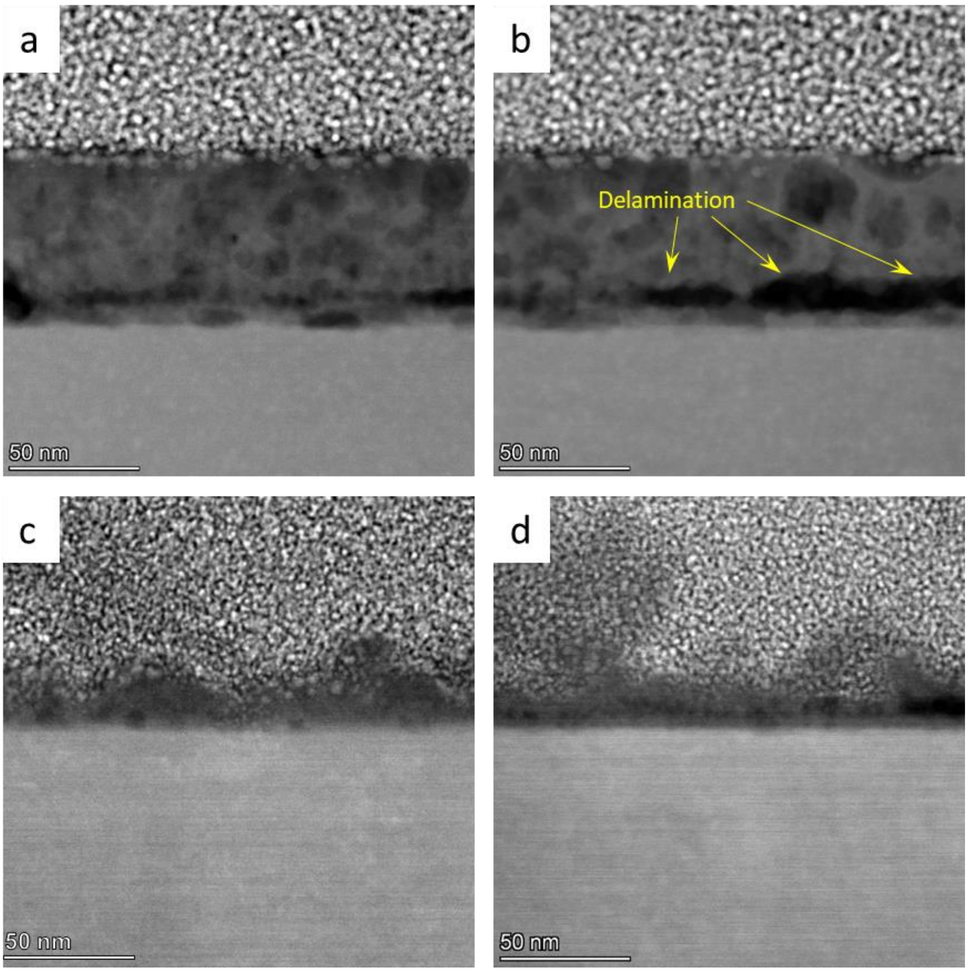

Figure S2. STEM images of cross-sectional specimen obtained from ZnS film, deposited from (a,b) $ZnSO_4$ and (c,d) $ZnCl_2$ solution after 5 min of deposition (a,c) before and (b,d) after 200 KeV electron beam irradiation for 10 min.

Table S1. Standard enthalpy of formation used in Figure 11 for the various Zn^{2+} salts used in this work.[1]

Precursor	ΔH_f^{298}, kJ/mol
Zn $(OC_2H3)_2$	-1671.1
$Zn(NO3)_2$	-483.7
$ZnCl_2$	-415.1
$ZnSO_4$	-980.1
ZnI_2	-208.2

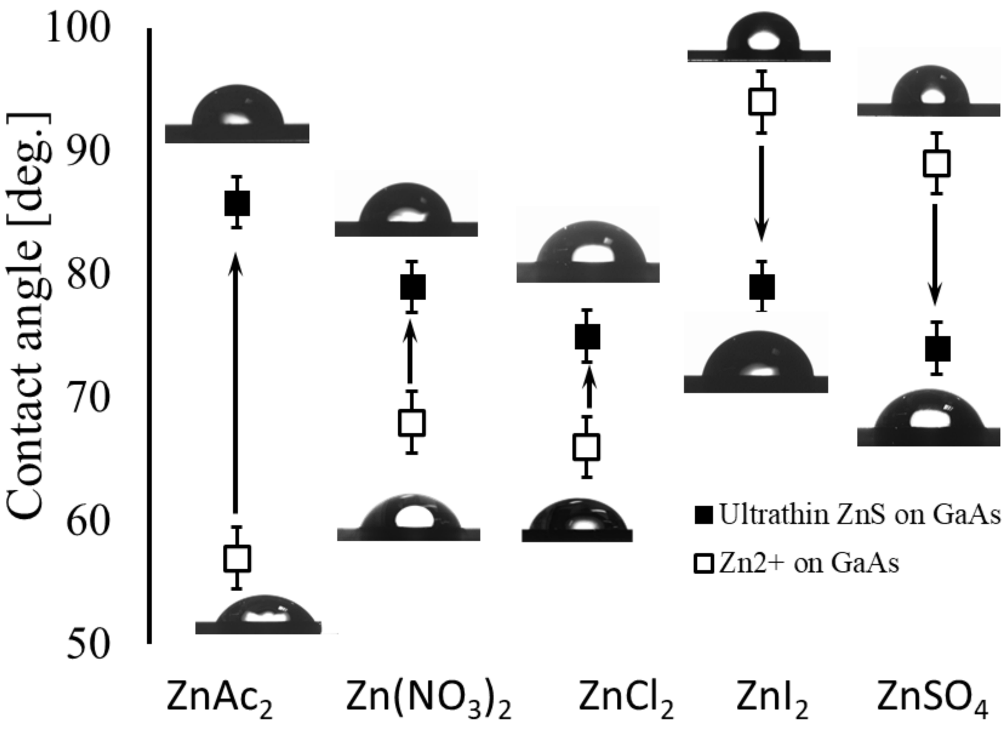

Figure S3. Contact angle measurement for ZnS films grown for 2 min from various precursors and GaAs substrates treated in Zn precursor solutions.

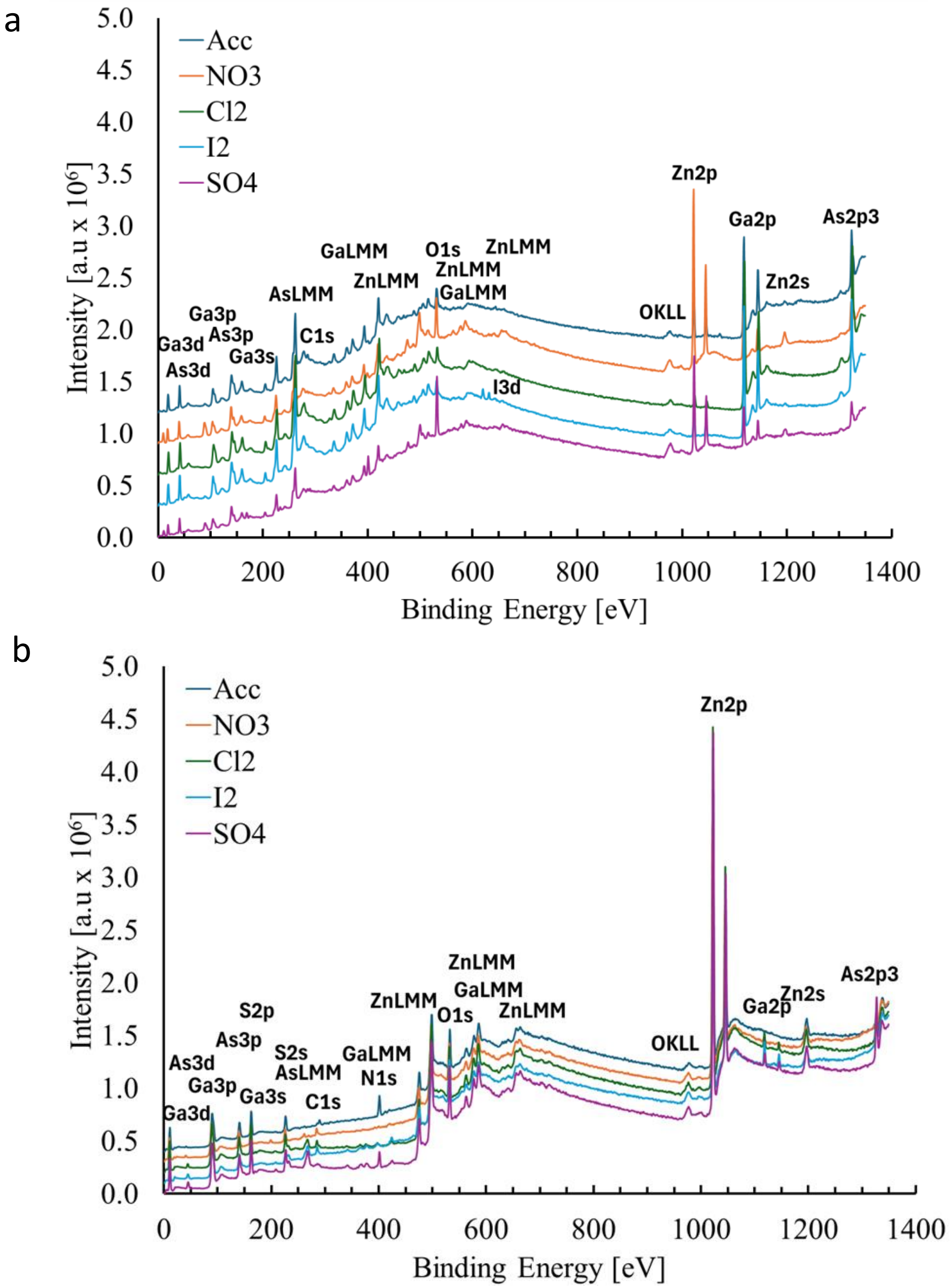

Figure S4. XPS survey spectra of a) GaAs treated in the solutions of various Zn precursors and b) of ZnS thin films after 5 min of deposition. Spectra are normalized to the total XPS signal obtained for each sample and shifted along Y axis for clarity
